# Reply to a G.-U. Critic

**Published:** 1902-04

**Authors:** Winfield Ayres

**Affiliations:** Genito-Urinary Surgeon, Bellevue Hospital, O. D. P., New York; Instructor in Genito-Urinary Diseases in New York University and Bellevue Hospital Medical College; Instructor in Genito-Urinary Diseases in the New York Post-Graduate Hospital, Etc.


### TEXAS MEDICAL JOURNAL.

ESTABLISHED JULY, 1885.

PUBLISHED MONTHLY.—SUBSCRIPTION $1.00 A YEAR.

VOL. XVII. AUSTIN, APRIL, 1902. No. 10.

### Original Contributions.

For Texas Medical Journal.

### Reply to a G.-U. Critic.

BY WINFIELD AYRES, M. D.,

Genito-Urinary Surgeon, Bellevue Hospital, O. D. P., New York; Instructor in Genito-Urinary Diseases in New York University and Bellevue Hospital Medical College; Instructor in Genito-Urinary Diseases in the New York Post-Graduate Hospital, Etc.

There are some critics, more common in the lay than in the medical press, though not of late confined to it, who seem to think more of themselves than of the subject they are discussing, or of the unfortunate victim they have under the scalpel, and who never appear so happy as when they are brandishing the tomahawk with a view to the popular applause. As a rule, they have a fine, breezy Western style, and their lucubrations are always extremely readable, whatever else they may be. They hold, with Sydney Smith, that it is a mistake to read a book (or a pamphlet) before reviewing it, on the ground that it gives a fellow such a confounded prejudice. When facts are in their way they brush them aside—everything must be sacrificed to the smartness of the critique and the consequent fame of its author.

With all respect to Dr. Frank Lydston, the distinguished G.-U. specialist of Chicago, I would take the liberty of suggesting that he is rapidly qualifying for the position of grand chief of the new literary order of Rough Riders, of whom I am speaking; and as proof thereof I would invite the attention of those interested to a contribution of his to the TEXAS MEDICAL JOURNAL (February, 1902), in which he indulges in a delightfully candid and otherwise characteristic criticism of a paper from my pen, entiled "The Treatment of Syphilis, with Especial Reference to the Best Methods of Administering Mercury," which was published in the London *Lancet* of October 19, 1901. From a literary point of view, the article—of course I mean Dr. Lydston's, not my own—was one of the most delightful it has been my pleasure to read for some time; but as a piece of criticism, I must deferentially submit that it was not quite fair either to me or the preparation on which I was making a report.

To begin with, I have to complain that Dr. Lydston has based his criticisms on an abstract of my paper instead of on the paper itself, though I should have thought the *Lancet* not unavailable in one of the public libraries of Chicago (if not on the doctor's own table), and the trouble of consulting the original might not be too much to expect of a gentleman so much interested in the subject as to be prepared to sit down ready to tear the article to pieces. Had the doctor done this, it would have saved him from some mistakes and gratuitous assumptions, as, for example, the one he makes in his opening remarks, "The paper is so manifestly devoted to the exploitation of Mercurol, a supply of which was evidently left at Bellevue Hospital for 'testimonial' experimentation by the manufacturers, that it possibly should not be taken seriously." Now, I have written two reports on Mercurol in the treatment of syphilis, one published in the *Philadelphia Medical Journal* ("The Treatment of Syphilis—A New and Tolerable Form of Administering Mercury, with Report of Sixty-five Cases Treated at Bellevue Hospital"), November 10, 1900, and the other in the London *Lancet* ("The Treatment of Syphilis, with Special Reference to the Best Methods of Administering Mercury"), October 19, 1901, and I thought I had made it sufficiently clear in each that whatever blame or credit was involved in the use of this preparation in the connection indicated was entirely mine. Mercurol, as doubtless Dr. Ldyston is aware, was originally introduced to the profession as a gonorrheal remedy. It came from Germany with the imprimatur of Dr. Karl Schwickerath, and accompanied by full details of the formula by which it was prepared. It was used experimentally and with success by Professor Ramon Guiteras, Dr. Valentine, and other prominent men here and in other parts of the country, who reported more or less favorably on it in the treatment of urethritis. It occurred to me that the combination of nucleol with mercury, of which Mercurol consisted, should make it an ideal form for administering mercury in syphilis, and, having procured a quantity of the drug, I had it made up for me in tablet form by my own chemists, Messrs. Bendiner and Schlesinger, of this city. All this was pretty fully explained in my first paper, and I now see that it would have been well if I had repeated the explanation in my second.

The facts just stated will show Dr. Lydston the injustice he has done both me and the manufacturers by his altogether unwarranted insinuation that we had entered into the unethical arrangement of making "testimonial" experiments. I do not know what is the custom in the hospitals in Chicago with which Dr. Lydston is connected, but I may inform him that at Bellevue we are not in the habit of showing much respect to medicaments that are dumped at our doors in the way suggested, nor yet for the manufacturers who would so dump them. In fact, so well do the indiscriminate distributors of samples know the fate awaiting them at all well-conducted New York hospitals that we now seldom or never see them. The nuisance has been allowed to cure itself. Mercurol, in any case, required no such introduction. It had come to us through regular and reputable channels, we knew positively what it was, it had stood the test of clinical use in the purpose for which it was originally intended—(so far the main objections I have heard to it as a gonorrheal remedy are that it is more expensive than some other preparations, and that its want of stability makes it important to have fresh solutions made at frequent intervals)—and I, of my own motive, and without any interest save that of my clients, adopted it experimentally for another form of disease in which its ingredients seem to show that it was indicated. Was there anything in this action of mine for which I am to be visited with censure? Dr. Lydston says my paper was "manifestly devoted to the exploitation of Mercurol." Assuredly it was, if by this is meant that I intended to convey to my fellow practitioners the belief that in it I had found what we had all long been looking for, "a new and tolerable form of administering mercury," and that as a consequence I would like them also to give it a trial. As regards the manufacturers of the preparation, it was only after my favorable reports, and perhaps because of my enthusiastic solicitation, that they were induced to put the tablets on their list, and now I am pleased to see that other specialists are using them, and obtaining equally good results with myself.

I frankly admit that the cases referred to in my paper were not as fully reported as I could have wished. This was due to lack of room in my clinic and an over supply of material. At that time I was treating at Bellevue an average of ninety patients a day; my room was fifteen feet square, and I was obliged to get through my work in two hours. Considering the extent to which I was handicapped, the records may be allowed to be fairly satisfactory, and even if they had been given in more detail they would not have been found to affect the general conclusions I drew. Of course, there are many points on which practitioners will differ. For example, I notice that Dr. Lydston, in his well known book, bars alcohol and tobacco. I agree with him as to alcohol, but in regard to tobacco, I prefer the patient who has been in the habit of using it to continue to do so to a moderate extent, and for this reason that I wish to know whether my patient is getting sufficient mercury to control the disease. The irritation of tobacco smoking will cause the symptoms to appear in the mouth quickly, and if such a slight cause is sufficient to produce mucous patches it shows that sufficient medicine is not being taken. As to the question of dosage generally, Dr. Lydston unquestionably knows that by "full dosage" I mean sufficient mercury to control the disease, and yet produce no untoward effects. That can be determined by no rule whatever; the expedient of decreasing the dose one-third from the point of tolerance certainly does not apply to all cases.

I come to another point of difference between us. Dr. Lydston sticks to protoiodide. I am surprised at the fact; but the younger men in the profession are now thinking for themselves, instead of accepting without question the teaching of brilliant writers, and the majority of them, so far as my experience goes, are discarding that form of mercury. I agree with the doctor that the effects of protoiodide are remarkable in many cases, but as frequently as not they are only remarkable in the sense of being satisfactory, for the first month or two. Then the medicine ceases to be either efficacious or tolerable, and some other drug has to be substituted. Now it is impossible in the treatment of syphilis, when it is thus found necessary to change off to another drug, to do otherwise than start with the smallest dose, go up to the limit and feel one's way back again, in order to ascertain the correct dosage, and in this way much valuable time is lost.

The doctor, however, says further: "The majority of syphilographers will admit that the protoiodide frequently shows marked control of active syphilis within one week, and shows a decided curative influence in most cases within two or three weeks." To this crushing argument, I might reply by reminding the doctor of the familiar fact that in some cases the roseola will disappear within a week without any medicine whatever.

Here I may remark that Dr. Lydston uses the word "control" in an entirely different sense from what I did in my paper. By control I mean absolute disappearance of the symptoms with the prospect that they will not return. What he means by the word, as I understand it, is the beginning of the subsidence of the active symptoms.

Another point I may touch on in passing is this: The doctor says of bichloride that it is notoriously unreliable. Yet he uses it in hypodermic injections. He must be a wizard to be able to find a drug that is reliable for hypodermic purposes and unreliable for internal administration. Again I find the remark that biniodide is also notoriously unreliable "save in the nascent form." To give nascent biniodide of mercury the patient must either prepare his medicine just before taking, or he must take the mercury separately from the iodine, and leave the biniodide to be formed in the stomach. For my own information, I really wish that the doctor would give us his method of using "nascent biniodide."

Before leaving this branch of the subject, I should like to say one or two words more in regard to bichloride. With the exception of the time I have been experimenting with Mercurol, the routine treatment of syphilis at my clinic at Bellevue has consisted of the internal use of bichloride. In severe cases we give hypodermatic injections, and in some cases inunctions; and my experience has been that it matters not how the mercury is administered there are some cases which it will not control unless you also give small doses of potassium iodide. And yet the doctor says potassium iodide should not be given in the secondary stage! What, I should like to ask, are we to do? Shall we keep on our doses of mercury until the tertiary symptoms appear, and then commence giving iodide? Really, we must have more precise instruction from those who go in for dogmatic teaching, and seem to think that all is known that can be learned in regard to the treatment of this elusive and so persistently recurrent disease. The doctor criticises the looseness of my reports, and in some respects, as I have admitted, with justice; but for a man of his extensive experience and national reputation to fall into this slip-shod method of criticism is indeed to be lamented. The virulence of his attack required something more than has been adduced to give it what an Irish friend of mine calls a "reasonable *raison d'etre.*" In what way, I am exceedingly curious to know, have I succeeded in working up the eloquent doctor to such a fine frenzy? Surely, the fact that I am an humble worker in New York, while he is the recognized authority of Chicago and the boundless West, is not sufficient to account for the zeal with which he has fallen upon me. From what I have heard of the geniality of my opponent's character, I am rather disposed to think he has rushed into print for the sheer love of the thing, as others do into a fight. In that case, I have no doubt we will understand one another better later on. Meanwhile, we must take care that no unintentional injustice is done either to individuals or to medical science; and, therefore, I would like to say a few more words on the subject matter of our controversy.

Among those who have followed my example in using Mercurol in the treatment of syphilis is my friend, Dr. Eugene Carson Hay, of Hot Springs, Ark., a place sometimes described as the Mecca of American syphilitics, and certainly a good field for observing the effects of such a remedy as the one under consideration. Dr. Hay publishes an interesting article in the *Charlotte Medical Journal* ("Modern Methods of Treating Syphilis") of February, 1902, and also read before the annual meeting of the Tri-State Medical Society, Memphis, Tenn., November 19, 1901. Perhaps he, also, will be accused of wishing to exploit the preparation, but in any case, I would advise Dr. Lydstone to read what he has to say. His concluding remarks are as follows:

"Of course it may be objected that one year's experience is not sufficient on which to base an opinion of the permanent effects of any drug used in the treatment of syphilis. This is perfectly true, but in any case the first thing to do is to control the symptoms, and that can apparently be done more satisfactorily with Mercurol than with any other preparation of mercury given internally. Besides, there is this to be borne in mind: We are not dealing with a new drug, but only with a new form of administering it. There is certainly no reason for believing that mercury given in the form of a nucleid will produce results of a less durable character than mercury given in any other form. While we can never say that syphilis has been completely extirpated, I feel there is some ground for satisfaction in being able, as I am, to say that out of the hundred patients who have been treated by me with Mercurol and baths only, and have been under treatment for a period of three months or longer, none show any trace of the disease except possibly a slight general adenopathy, and those under my care are getting along as well as could be desired."

Here is a modest, conservative report, not dissimilar, as I claim, in character to my own, and bearing out my conclusions as far as they have gone. I may here add that some experiments I have conducted on the blood seem to show that the nuclein in the combination has a direct cell-forming action. However, these experiments have not been concluded, and I am not prepared to make any positive declaration on this point. All that I do is to say, as Dr. Hay says,—and now I have had nearly two years' experience with the preparation,—that Mercurol controls the symptoms better than any other form of mercury.

Dr. Lydston has not said anything against the drug itself. All he does is to find fault with the form of my report, and to bring all his artillery to bear against me on this account, as if I had committed some heinous offense. Perhaps he will say that he desired to protect the practitioner; but, as I said a little earlier, the younger men in the profession are beginning to think for themselves. They no longer accept the *ipse dixit* of others in regard to drugs or treatment generally; consequently, there is little danger of either my critic or myself leading them astray. All I did was to lay the results of my experience before my fellow workers in order that they might compare them with their own. This I propose to continue to do, knowing that what I do in this regard will be appreciated, as I appreciate that done in the same spirit by others. Dr. Lydston gives me credit for honesty. I thank him sincerely, but as the inference is that I must be equally characterized by simplicity, if not more so, the measure of my gratitude to him even on this point need not be large. I think, however, I have said enough to show the candid reader that, whether right or not, I have at all events not been imposed upon by any designing trader. Aspersions of this kind are too freely flung about, and perhaps they are too commonly deserved.

Let me say just a word in conclusion in regard to Mercurol. My latest experience—or rather all my experience down to the latest time—convinces me that, though it is not a drug that will control every case, it is the most reliable and most easily borne preparation of mercruy that I have ever used. I want to emphasize the fact that Mercurol must not be given in solution, because it is not stable, and changes to the oxide quickly. This fact, though touched on in my paper, is not mentioned in any of the abstracts that I have seen, and it is a matter of considerable importance. I make a point of giving the medicine in the form of tablets, commencing with one grain t. i. d., and adding one more grain per day every fourth day, until there is slight salivation, or slight looseness of the bowels, or there begins to be some improvement in the patient. It is essential that the increase should be slow because every grain of Mercurol contains one-tenth of a grain of pure mercury, which, of course, is a large dose.

I shall probably report later on the full results of my investigations into the hematological part of the subject. Meanwhile, I see no reason for changing the conclusion at which I formerly arrived: that it is the most tolerable and otherwise best means of administering mercury with which we are acquainted.

